# RuleMonkey: software for stochastic simulation of rule-based models

**DOI:** 10.1186/1471-2105-11-404

**Published:** 2010-07-30

**Authors:** Joshua Colvin, Michael I Monine, Ryan N Gutenkunst, William S Hlavacek, Daniel D Von Hoff, Richard G Posner

**Affiliations:** 1 Clinical Translational Research Division, Translational Genomics Research Institute, Phoenix, AZ 85004, USA; 2 Theoretical Biology and Biophysics Group, Theoretical Division and Center for Nonlinear Studies, Los Alamos National Laboratory, Los Alamos, NM 87545, USA; 3 Department of Biology, University of New Mexico, Albuquerque, NM 87131, USA; 4 Department of Biological Sciences, Northern Arizona University, Flagstaff, AZ 86011, USA

## Abstract

**Background:**

The system-level dynamics of many molecular interactions, particularly protein-protein interactions, can be conveniently represented using reaction rules, which can be specified using model-specification languages, such as the BioNetGen language (BNGL). A set of rules implicitly defines a (bio)chemical reaction network. The reaction network implied by a set of rules is often very large, and as a result, generation of the network implied by rules tends to be computationally expensive. Moreover, the cost of many commonly used methods for simulating network dynamics is a function of network size. Together these factors have limited application of the rule-based modeling approach. Recently, several methods for simulating rule-based models have been developed that avoid the expensive step of network generation. The cost of these "network-free" simulation methods is independent of the number of reactions implied by rules. Software implementing such methods is now needed for the simulation and analysis of rule-based models of biochemical systems.

**Results:**

Here, we present a software tool called RuleMonkey, which implements a network-free method for simulation of rule-based models that is similar to Gillespie's method. The method is suitable for rule-based models that can be encoded in BNGL, including models with rules that have global application conditions, such as rules for intramolecular association reactions. In addition, the method is rejection free, unlike other network-free methods that introduce null events, i.e., steps in the simulation procedure that do not change the state of the reaction system being simulated. We verify that RuleMonkey produces correct simulation results, and we compare its performance against DYNSTOC, another BNGL-compliant tool for network-free simulation of rule-based models. We also compare RuleMonkey against problem-specific codes implementing network-free simulation methods.

**Conclusions:**

RuleMonkey enables the simulation of rule-based models for which the underlying reaction networks are large. It is typically faster than DYNSTOC for benchmark problems that we have examined. RuleMonkey is freely available as a stand-alone application http://public.tgen.org/rulemonkey. It is also available as a simulation engine within GetBonNie, a web-based environment for building, analyzing and sharing rule-based models.

## Background

### Introduction

A great deal of knowledge about signal transduction, which is mediated largely by the interactions of proteins, has been built up over the years [1,2], in part because molecular changes that affect signal-transduction systems play a role in many diseases, such as cancer [3]. Signal-transduction systems are exceedingly complex, and as a result, our ability to manipulate the behaviors of these systems (e.g., through therapies based on molecularly targeted drugs [4]) is limited. To extend our understanding beyond that reachable through intuition alone, researchers are attempting to develop predictive mathematical models for signal-transduction systems [5-7]. These systems are difficult to model for a variety of reasons [8], and new modeling approaches, such as rule-based modeling [9,10], are needed. Rule-based modeling is notable because it provides a solution to the problem of combinatorial complexity [11].

Central to rule-based modeling is the concept of reaction rules, which will be elaborated in detail below. One can think of a rule as a generalized reaction or a coarse-grained description of a molecular interaction and its consequences. The power of a rule is that it can concisely capture the site-specific details and consequences of an interaction of a structured molecule, such as a protein [9,12]. Functionally, a rule defines 1) the necessary and sufficient properties of reactants in a class of reactions arising from a molecular interaction, 2) the products of a reaction in this class given any particular set of reactants, and 3) a rate law that governs all reactions defined by the rule.

A set of rules for the protein interactions in a signal-transduction system typically implies a much larger set of reactions. The reason is that processes such as multivalent binding and multisite phosphorylation can yield a combinatorial number of chemically distinct protein complexes and phosphoforms [11,13]. The sheer size of such networks, as well as the cost of simulating large-scale chemical reaction networks using conventional methods, has posed a formidable barrier to the development and analysis of models for signal-transduction systems that account for site-specific details of protein interactions (in terms of rules).

To address the problem of simulating models defined by rules that imply large-scale (bio)chemical reaction networks, Colvin et al. [14] generalized the stochastic simulation method of STOCHSIM[15,16] and implemented this method in software called DYNSTOC, which is freely available [17]. The STOCHSIM/DYNSTOC simulation method involves the use of rules to determine if randomly selected components of molecules undergo a reaction. The method has a computational cost that is independent of the size of the reaction network implied by a set of rules. In this respect, the method is similar to methods reported by Danos et al. [18], Yang et al. [19], and Yang and Hlavacek [20]. These methods have been called "network-free" methods [21], in part because the efficiency of these methods is independent of the size of the reaction network implied by rules.

A key feature of DYNSTOC is its ability to simulate models encoded in the BioNetGen language (BNGL), a general-purpose model-specification language that has been described in detail and that can be interpreted by a number of software tools [21]. Thus, DYNSTOC can be used to simulate a wide array of rule sets that imply large-scale chemical reaction networks. Unfortunately, the network-free simulation method implemented in DYNSTOC is a null-event stochastic simulation method, and as a result, simulation of models with fast reactions is inefficient, because the size of the fixed time step used in the simulation procedure is determined by the rate of the fastest reaction.

Here, we present a software tool, RuleMonkey, which implements a network-free stochastic simulation method for determining the system-level dynamics of molecular interactions represented by rules. In this method, unlike in the null-event method of STOCHSIM/DYNSTOC, the size of the time step is variable, and each time step results in a change of the state of the system being simulated. Like DYNSTOC, RuleMonkey is capable of simulating models defined using BNGL.

### Overview of graphical formalism underlying BNGL

The algorithm presented below is intended for simulation of rule-based models that can be specified using BNGL [21], particularly models for the system-level dynamics of protein-protein interactions in signal-transduction systems. In such a model (for examples, see [22-24]), molecules and molecular complexes are represented as graphs, and molecular interactions are represented as graph-rewriting rules. It will be useful to briefly review the graphical formalism underlying BNGL [21,25-27].

Molecules (e.g., proteins) are taken to be the building blocks of chemical species (e.g., protein complexes) and to be composed of reactive components, such as amino acid residues subject to post-translational modifications, linear motifs (e.g., proline-rich sequences of the form PxxP), and protein interaction domains [e.g., Src homology 3 (SH3) domains]. Thus, in a rule-based model, molecules and their components comprise a set of chemical species *S *= {*S*_1_, *S*_2_, ...}, and each species *S_i _*is represented by a graph *G_i _*= (*V*_*i*_, *E*_*i*_), where the vertices *V_i _*represent molecular components and the edges *E_i _*represent (non-covalent) bonds between components. Thus, the connectivity of molecules within a chemical species is explicitly represented, and the components that mediate molecular interactions are identified. The vertices of a graph are labeled. By convention, vertices that share the same label are taken to be chemically indistinguishable. Labels are immutable. In addition to labels, vertices can be associated with mutable attributes, which can be used to represent the (time-dependent) internal states of components. It is often convenient to introduce an internal state to represent the phosphorylation status of an amino acid residue [25]. By introducing an internal state for each amino acid residue (S/T/Y) subject to modification by kinases and phosphatases, one can track all possible phosphoforms of a protein.

Rules are used to implicitly represent reactions arising from molecular interactions. A rule *g*_LHS _→ *g*_RHS _is composed of 1) sets of left-hand side (LHS) and right-hand side (RHS) pattern graphs, *g*_LHS _and *g*_RHS_, which can be viewed as subgraphs of the graphs used to represent chemical species or species graphs, which are denoted *G *= {*G*_1_, *G*_2_, ...}; 2) a mapping of the vertices of LHS pattern graphs to the vertices of RHS pattern graphs, which defines a graph transformation; 3) a rate law; and 4) application conditions.

Application conditions are optional but almost all rules encoded in BNGL are endowed with an application condition that restricts molecularity [21,27]. A particular chemical species is a reactant in a rule-defined reaction if its corresponding species graph is matched by a LHS pattern graph (i.e., the species graph contains a subgraph that is isomorphic to the LHS pattern graph) and, in addition, it and any potential co-reactant satisfy the application conditions associated with the rule (if any). The set of molecular components (vertices) directly affected by a transformation will be called a reaction center. Because many species graphs can potentially be matched by a single LHS pattern graph, a rule generally defines not a single reaction, but an entire class of reactions, all of which involve a common transformation. This transformation affects the same reaction center but can take place in multiple molecular contexts, depending on the LHS pattern graph(s) of the rule and the application conditions (if any) of the rule.

Rules are typically associated with application conditions that impose constraints on the molecularity of reactions and the number of reaction products [19,27]. In BNGL, separation of two LHS pattern graphs by a "+" symbol indicates a molecularity of 2, and a rule with a single LHS pattern graph indicates a molecularity of 1. Similarly, separation of two RHS pattern graphs by a "+" symbol indicates two reaction products, and a single RHS pattern graph indicates a single reaction product. Application conditions can be introduced for a variety of other reasons. For example, Monine et al. [28] recently modeled steric effects on multivalent ligand-receptor binding using rules with application conditions that place constraints on the allowable geometric configurations of ligand-induced receptor aggregates. Here, we will only consider application conditions related to molecularity and number of reaction products.

Above, we have implicitly assumed that rules define unidirectional reactions. In BNGL, a short hand notation (< - >) can be used to indicate that the pattern graphs of a rule can be reversed to define reverse reactions [21]. This notation is convenient, as it allows one to avoid redundancy in a model specification. However, the distinction between LHS and RHS graphs is obfuscated. In the discussion that follows, for clarity, we will continue to assume that rules define unidirectional reactions only, which is equivalent to assuming that only the - > notation within BNGL is available. From this point of view, two rules are required to define reversible transformations. For example, A - > B and B - > A must be used instead of simply A <-> B where A and B are pattern graphs. In short, we will refer to those graphs in rules that are used to identify reactants as LHS pattern graphs and those graphs that are used to define transformations as RHS pattern graphs.

The advantage of the rule-based modeling formalism is that a modeler can use a rule to concisely and comprehensivley account for the site-specific details and consequences of a molecular interaction, no matter how many distinct reactions might arise from the interaction. However, this expressiveness comes at a cost. Each individual reaction defined by a rule is assigned the same rate law up to a statistical factor, which can vary from reaction to reaction within a reaction class [21,27]. (Statistical factors are discussed further below.) In reality, each reaction in a reaction class may be characterized by a unique rate law. Thus, the rate law associated with a rule provides only a coarse-grained description of the kinetics of the reactions within the rule-defined reaction class. However, because a rule can specify the molecular context that is permissive for reaction, the coarseness of a rule can be adjusted by tuning the contextual elements of the rule. At the finest level, the contextual elements are highly specific and a rule defines a unique reaction. At the coarsest level, the rule is free of contextual constraints, meaning that the rule indicates that a reaction center can undergo a reaction regardless of the molecular context in which that reaction center is found.

### Model assumptions

Having presented an overview of the graphical formalism underlying BNGL, we can now introduce the assumptions upon which the algorithm implemented in RuleMonkey is based.

We consider a well-mixed isothermal reaction compartment of volume *V *containing a set of *M *molecules *P *= {*P*_1_, ..., *P*_*M*_}, which we take to be proteins. Each molecule *P*_*i *_is associated with a set of components *c*_*i *_= {*c*_*i*1_, *c*_*i*2_, ...}. These components, each of which are optionally associated with an internal state, undergo reactions according to a set of *N *reaction rules *R *= {*R*_1_, ..., *R*_*N*_}. Each rule *R*_*i *_defines a class of reactions, and each member of this class is characterized by a single rate law up to a statistical factor, as mentioned earlier. Given this rate law, a cumulative rate *r*_*i *_can be determined for the class of reactions defined by *R*_*i*_, at least in principle. This cumulative rate corresponds to the sum of the rates for the individual reactions in the class of reactions defined by *R*_*i*_. By convention, the rate of each reaction will be taken to be given in terms of the number of molecules undergoing the reaction per unit time in *V*. In other words, each *r*_*i *_has the same units as 1*/t*.

As a simplification, we will consider only mass-action rate laws. Thus, for example, we will assume that a bimolecular reaction *A *+ *B *→ product(s) is characterized by a rate law of the form *fk_V _n_A_n_B_*, where *n_A _*is the number of *A *type molecules or molecular complexes in *V *, *n_B _*is the number of *B *type molecules or molecular complexes in *V *, *k_V _*is a rate constant expressed in the same units as 1*/t*, and *f *is a statistical factor that accounts for the number of (chemically indistinguishable) ways that the *A *and *B *molecules (or molecular complexes) can react, i.e., the number of degenerate reaction paths from reactants to products. These molecules (complexes) can react in more than one way if, for example, *A *and *B *associate via binding sites that are present in *A *and/or *B *in multiple copies. Thus, *k_V _*is a so-called "single-site" rate constant. Rate constants associated with rules are assumed to be single-site rate constants according to the conventions of BNGL [21]. Note that *k_V _*is volume dependent and can be equated to *k/*(*N_A_V *), where *N_A _*is Avogadro's number and *k *is the rate constant expressed in the same units as *N_A_V/t*. The statistical factor *f*, which is related to symmetry, can be determined as described by Blinov et al. [27].

As another simplification, we will assume that internal-state dynamics are described by two-state trajectories. Thus, a component *c_ij _*of molecule *P_i _*will be associated with an internal state *σ_ij_*(*t*) ∈ 0[1] or none at all. (Recall that a component need not be associated with an internal state.) If a component is a tyrosine residue subject to phosphorylation and dephosphorylation, then an internal state could be introduced to represent its phosphorylation status [25], with one state value representing the unphosphorylated form and the other state value representing the phosphorylated form. Software tools for rule-based modeling, including RuleMonkey, actually allow for greater than two internal states to be associated with a component.

For purposes of discussion, we will focus on rules specifying reactions that result in the association or dissociation of two components or that change the internal state of a component (i.e., reactions that can be modeled as graph transformations involving the addition or removal of an edge in a graph or the change of a vertex attribute in a graph). Note that a component is taken to have at most one binding partner at a time, as usual [21]. Extension of the discussion that follows to account for other types of rules, such as rules for synthesis, degradation and trafficking between compartments, is straightforward. RuleMonkey is capable of handling these types of reactions, which can be specified in accordance with the conventions of BNGL [21]. For a system in which only association, dissociation, and state-change reactions are possible, counts of molecules and components are conserved quantities. Moreover, for such a system, the LHS pattern graph(s) of a rule will serve to identify either one or two reactants for each reaction defined by the rule.

### The population-based approach to simulation of a rule-based model

The state of a system governed by a rule set *R *can be taken to be given by the vector **x**(*t*) = (*x*_1_(*t*), ..., *x_n_*(*t*)]*^T^*, is the population level of the molecule or molecular complex *S_i _*described by graph *G_i _*and *n *is the number of chemical species that are populated over the time interval of interest. In the limit of continuous population sizes, x(*t*) evolves deterministically according to a system of coupled ordinary differential equations (ODEs):(1)

where the vector field **f**(**x**) is composed of mass-action terms derived from the rate laws associated with the rule set *R*. For a rule set that implies *m *unidirectional reactions, there are *m *mass-action terms.

In principle, Eq. 1 can be derived from *R *[25,29]. However, derivation and numerical integration of Eq. 1 is impractical when a rule set implies a large-scale chemical reaction network (i.e., large *m *and *n*), which is often the case [9,14,18,19]. Reduced forms of Eq. 1, involving transformations of the variables x, can sometimes be found through analysis of *R *[30-34], and on-the-fly stochastic simulation procedures [25,35,36] can sometimes be used to limit enumeration of the reactions implied by *R*, which is expensive. Nevertheless, these methods fail to be practical for many rule sets.

To overcome this problem, kinetic Monte Carlo (KMC) procedures have been developed in which *R *is used directly to generate stochastic trajectories consistent with the chemical master equation that corresponds to Eq. 1 [14,18-20]. These procedures avoid enumerating the reaction network implied by *R*. Thus, the term "network-free" is apt for these simulation procedures. The algorithm presented below is a network-free procedure.

### The particle-based approach to simulation of a rule-based model

The RuleMonkey algorithm, like other network-free simulation procedures, is particle based, meaning that each and every material component of a system is tracked, regardless of whether the material components are chemically distinguishable. In other words, given a set of rules *R *and a set of instances of chemical species graphs, each potentially present in multiple copies, these graphs and their constituent parts are tracked individually as the graph transformations defined by *R *are applied to them (as part of a simulation procedure) and the constituent parts move through the space of possible chemical species. Let us use  to denote the set of instances of chemical species in a system of volume *V *at time *t*. These instances of chemical species can, in principle, be partitioned into disjoint sets, the set of *n *populated chemical species:(2)

where  is the *j*th copy of species *S_i _*and *x_i_*(*t*) is the population level of (or equivalently, the number of copies of) species *S_i _*at time *t *in *V *. In a network-free simulation procedure, the species instances  are tracked but they are not partitioned as indicated in Eq. 2, which is essentially equivalent to determining x(*t*), the population levels of the chemical species in *V*. To determine x(*t*) from , one must identify and count the graphs in  corresponding to each species in *S*(*t*), which is an expensive procedure because it necessarily involves repeated solution of the graph isomorphism problem [21,27].

Instead of relying on a partition of  as indicated in Eq. 2 or knowledge of x(*t*) to advance a simulation, a network-free algorithm relies on knowledge of the complete component state of a system, i.e., the state of each component in the system. The state of an individual component *c *is the information required to uniquely identify 1) the component, including its label and the label of the molecule of which it is a part; 2) the internal state of the component (if any); and 3) the bound state of the component, including its binding partner (if any). Let us use ∑*_c_*(*t*) to denote the complete component state of a system, which we will also refer to as simply the system state. Note that ∑*_c_*(*t*) fully defines  (and vice versa). Because the graph-rewriting operations defined by rules directly alter ∑*_c_*, the system state as expressed in terms of component states can be easily tracked (with a memory requirement that depends on the number of material components in *V *) and ∑*_c_*(*t*) can be dynamically updated as rules in *R *are applied to graphs in  in a simulation procedure.

#### Observables

There is a one-to-one relationship between ∑*_c_*(*t*) (or ) and x(*t*), but as indicated above, the RuleMonkey algorithm, like other network-free simulation procedures, avoids determining x(*t*). How then are system properties of interest determined as a function of time?

The output of a network-free simulation procedure is determined by a specification of a set of *ω *observables, *O *= {*O*_1_, ..., *O_ω_*}. Each observable *O_i _*is composed of a pattern graph *g_i_*, making it similar to a rule. It is also associated with a time-dependent match number *m_i_*(*t*), which is a function of *g_i _*and the system state:(3)(4)

and *w*(*g_i_*, *s*) is a weight that is specified to be either 1 or the number of times that *s *is matched by *g_i_*, i.e., the number of distinct images of *g_i _*in *s *or the number of distinct mappings (injections) of the elements of *g_i _*to a subset of the elements of *s*. In a BioNetGen input file, the former case is indicated by the BNGL keyword Species, whereas the latter case is indicated by the BNGL keyword Molecules [21]. The match number associated with an observable of the Species type is a count of the number of chemical species in *V *containing at least one moiety matched by the pattern graph of the observable. The match number associated with an observable of the Molecules type is a count of the number of moieties in *V *matched by the pattern graph of the observable. Because the match numbers associated with observables are functions of the system state (Eqs. 3), these quantities can be easily tracked and dynamically updated as the system state changes in a simulation procedure.

### Algorithm

Given the background presented above, we can now present an outline of the simulation procedure implemented in RuleMonkey. This procedure has more or less already been described in earlier work [18-20], although key implementation details, described below, are novel. The procedure can be used to produce stochastic trajectories consistent with the chemical master equation corresponding to a system of ODEs of the form of Eq. 1. A comparison of methods is included below.

We take as given a set of *N *rules *R *= {*R*', *R*"}, where *R*' denotes the set of rules with a single LHS pattern graph and *R*" denotes the set of rules with a pair of LHS pattern graphs. We also take as given an initial time *t*(= 0), a system volume *V *, and a set of initial species instances , from which the component state of the system, ∑*_c_*(*t*), can be determined. For now, we will assume that there is a procedure that allows us to calculate the set of rates *r*(*t*) = {*r*_1_(*t*), ..., *r_N_*(*t*)} associated with *R*. The method by which these rates are calculated will be described in detail below.

We will also assume that there are procedures that allow us to identify the potential reactants in rule-defined reactions and the reaction centers in these reactants. Thus, for a rule *R_u _*in *R*' we will assume that a set of species instances  can be identified as matching the LHS pattern graph of *R_u_*. The species instances in  are potential reactants in a reaction defined by *R_u_*. The reaction centers in  are the components that are matched by the LHS pattern graph of *R_u _*and that are directly modified in a reaction defined by *R_u _*(e.g., a component that joins or leaves a bond with another component or that changes internal state). Likewise, for a rule *R_v _*in *R*", we will assume that sets of species instances  and  can be identified that match the first and second LHS pattern graphs of *R_v_*, respectively. Pairs of species instances in  are potential co-reactants in a reaction defined by *R_v_*.

We can now outline the key steps of the network-free simulation procedure implemented in RuleMonkey, which is similar to Gillespie's method [36].

#### Initialize

Specify *V*, *t*, *R*, including the single-site rate constants in rate laws associated with rules, and a seed set of species instances , which determines ∑*_c_*(*t*). For each rule *R_i _*in *R*, calculate the rate *r_i _*associated with this rule and identify the species instances in  that are matched by the LHS pattern graph(s) of the rule. Finally, specify a set of observables, *O*, and use Eq. 3 to calculate the match numbers associated with these observables.

#### Increment time and select a rule in accordance with rule rates

Calculate*τ*, the waiting time to the next reaction event in the system, using(5)

where *ρ*_1 _∈ (0,1) is a uniform deviate and  Recall that the rule rate *r_i_*, which is taken to have units of inverse time in Eq. 5, is the cumulative rate of all possible reactions defined by rule *R_i_*. Equation 5 follows from , where *P*(*τ*) is the probability distribution function for *τ *[37]. This distribution holds if the rate at which the system leaves state ∑*_c_*(*t*) is simply *r*_tot_, the sum of the cumulative rates for the classes of reactions through which the system state can change.

Determine the class of the next reaction event by finding the smallest integer *I *that satisfies(6)

where *ρ*_2 _∈ (0,1) is a second uniform deviate. The next reaction event will be in the class defined by rule *R_I_*, where *I *∈ [1, ..., *N*]. Equation 6 indicates that the class of the next reaction event is randomly selected with probability in proportion to the cumulative rate of reactions within that reaction class. It should be noted that the cost of finding *R_I _*using Eq. 6 is proportional to *N*. A more efficient procedure, with cost proportional to log*N*, could be implemented [38,39], but Eq. 6 is used in RuleMonkey for simplicity. For simulation problems that we have considered, performance profiling indicates that this simplification does not limit the efficiency of RuleMonkey.

#### Select a set of reactants and use the selected reactant(s) and rule to change the system state

Additional uniform deviates are needed in the simulation of a reaction event. If *R_I _*∈ *R*' then a species instance  is randomly selected with probability in proportion to the number of distinct reaction centers in *s *matched by the LHS pattern graph of *R_I_*. On the other hand, if *R_I _*∈ *R*" then a first species instance  is randomly selected with probability in proportion to the number of distinct reaction centers in *s*_1 _matched by the first LHS pattern graph of *R_I _*, and a second species instance  is randomly selected with probability in proportion to the number of distinct reaction centers in *s*_2 _matched by the second LHS pattern graph of *R_I_*. The graph transformation defined in rule *R_I _*is then applied to a randomly selected set of reaction centers in the selected reactant(s), which changes the system state ∑*_c _*(or equivalently, ). In other words, the randomly selected set of species instances, *s *or {*s*_1_, *s*_2_}, is essentially removed from the system and replaced by a new set of species instances. The new species instance(s) correspond to the product(s) of the reaction event defined by *R_I_*.

#### Update sets and quantities that depend on the system state

Increment time *t *← +*τ *and update the sets of reactants associated with rules. Likewise, update the match numbers associated with observables. The steps outlined above that follow initialization are iterated until a stopping criterion is satisfied.

### Implementation

The simulation method described above has been implemented in software called RuleMonkey. RuleMonkey is available as a stand-alone application, which is freely available via the RuleMonkey web site [40], and as a simulation engine within the GetBonNie environment [41,42]. The input for a simulation is a BNGL-encoded model and simulation specification. In other words, RuleMonkey reads and interprets standard BioNetGen input files, like DYNSTOC [14] and BioNetGen [25,29,43]. The conventions of BNGL are described in detail elsewhere [21]. The novel details of algorithm implementation, which are related to the calculation of rule rates, are described below. Usage considerations are also discussed.

### Calculation of rule rates

**Figure 1:**
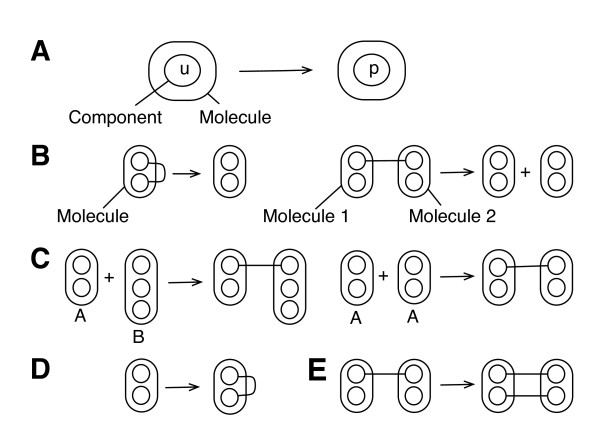
**Examples of reactions**. Five types of reactions are illustrated using the graphical conventions of Faeder et al. [26]: a) a state-change reaction; b) two dissociation reactions, one that results in a single product and one thatresults in two products; c) two bimolecular association reactions, one of the form *A *+ *B *→ product(s) and one of the form *A *+ *A *→ product(s); d) a monogamous ring-closure reaction; and e) a non-monogamous ring-closure reaction.

Below, equations are given for exactly calculating the rates associated with five types of reaction rules, which generate reactions of the types illustrated in Fig. 1. The five rule types are sufficient to specify a wide range of models. For efficiency, the equations given below are used only in the initialization step of the simulation algorithm. After initialization, as described in detail for rejection-free simulation of multivalent ligand-receptor interactions [20], the rates given by these equations are updated on the fly as reactions occur, which requires that matches of LHS pattern graphs in rules be tracked during a simulation.

#### Unimolecular state-change reactions

We will use *R_a _*to denote a rule that defines unimolecular state-change reactions, such as that illustrated in Fig. 1(a). We will assume that *R_a _*has the following form: *l*_*a *_→ *ρ *_*a*_, where *l_a _*is a LHS pattern graph and *ρ *_*a *_is a RHS pattern graph. Thus, *R_a _*defines unimolecular reactions that each have a single reactant and a single reaction product. We will assume that the pattern graph *l_a _*identifies a reaction center composed of a single molecular component in a particular internal state.

Given a set of species instances  at time *t*, the instantaneous rate *r_a_*(*t*) associated with *R_a _*is given by the following expression:(7)

where *k_a _*is the single-site rate constant associated with the mass-action rate law of *R_a _*and *v*_*a*_(*l*_*a*_, *s*) is a function that gives the number of distinct reaction centers in species instance *s *matched by *l_a _*(i.e., the number of components in species instance *s *in an internal state consistent with the component and internal state identified by *l_a_*). The function *v_a _*is similar to that of *μ *(Eq. 4). Note that *v_a_*(*l_a_, s*) = 0 if no subgraph of *s *is isomorphic to *l_a_*. Also note that *v*_*a*_(*l*_*a*_, *s*) is not necessarily equivalent to the number of times that *s *is matched by *l_a _*but rather is equivalent to the number of distinct reaction centers among the subgraphs of *s *matched by *l_a_*. It is important to make the distinction between number of matches and number of distinct reaction centers in matches when a LHS pattern graph specifies a contextual constraint that can be satisfied in more than one way.

In the course of a RuleMonkey simulation, rates of the form of *r_a _*are updated on the fly as reactions occur and are never recalculated *de novo *after initialization, which would be inefficient. Thus, for example, if a species instance *s *matched by *l_a _*is removed from , then *r_a _*is decremented by *k*_a_*v*_*a*_(*l*_*a*_, *s*). Similarly, if a species instance *s *matched by *l*_*a *_is added to , then *r*_*a *_is incremented by *k_a_v_a_*(*l_a_, s*). Updates of rule rates are facilitated by tracking matches of LHS pattern graphs of rules to subgraphs of the graphs representing the species instances in a system. As part of this bookkeeping procedure, when a new species instance appears in a system (as a result of reaction), it is checked against all the LHS pattern graphs of rules to determine the types of reactions in which it can potentially participate and to calculate updates of the rates associated with rules. When a species instance disappears from a system (as a result of reaction), the rates associated with rules are appropriately adjusted as well. The cost of updating a rate such as that given by Eq. 7, or any of the equations given below, depends on the number of rules in a model and the number of chemical species instances created and destroyed in a reaction. However, the cost per time step is more or less fixed over the course of a simulation. The overall efficiency of simulation depends on the efficiency of the procedures used to update rates and to store and update other information needed to implement the simulation algorithm described above.

#### Unimolecular dissociation reactions

We will use *R_b _*to denote a rule that defines unimolecular dissociation reactions, such as those illustrated in Fig. 1(b). We will assume that *R_b _*has one of two forms: *l_b _*→ *ρ_b _*or  where *l_b _*is a LHS pattern graph, and *ρb*, , and  are RHS pattern graphs. In the former case, *R_b _*defines unimolecular reactions that each have a single reaction product, as when a bond opens but no constituent parts of the reactant species dissociate. In the latter case, *R_b _*defines unimolecular reactions that each have two reaction products, as when a bond opens and constituent parts of the reactant species dissociate from each other. We will assume that the pattern graph *l_b _*identifies a reaction center composed of two molecular components that are directly connected by a (non-covalent) bond.

Given a set of species instances  at time *t*, the instantaneous rate *r_b_*(*t*) associated with *R_b _*is given by the following expression:(8)

where *k_b _*is the single-site rate constant associated with the mass-action rate law of *R_b _*and *v*_*b*_(*l*_*b*_, *s*) is a function that gives the number of distinct reaction centers in species instance *s *matched by *l*_*b *_(i.e., the number of pairs of connected components in species instance *s *identified by *l*_*b*_). Note that a species instance *s *is considered to be matched by *l_b _*only if application of the graph-rewriting operation of *R_b _*to *s *produces the correct number of reaction products, i.e., the number of products consistent with the RHS of *R_b_*. The function *v_b _*in Eq. 8 is analogous to the function *v_a _*in Eq. 7.

As noted above for rates of the form of *r_a_*, rates of the form of *r_b _*are updated on the fly as reactions occur during the course of a RuleMonkey simulation. These updates are straightforward and follow from Eq. 8.

#### Bimolecular association reactions

We will use *R_c _*to denote a rule that defines bimolecular association reactions, such as those illustrated in Fig. 1(c). We will assume that *R_c _*has the following form:  where *l_c _*and  are LHS pattern graphs and *ρ *_*c *_is a RHS pattern graph. We will assume that the pattern graphs *l_c _*and  identify two reactants and a single component within each. This pair of components forms the reaction center identified by *R_c _*and these components are connected in the product of reaction. Consistent with the conventions of BNGL [21], the plus sign that separates *l_c _*and  indicates that the molecularity of reactions defined by *R_c _*must be 2.

Given a set of species instances  at time *t*, the instantaneous rate *r_c_*(*t*) associated with *R_c _*is given by(9)

where(10)

and(11)

In the above equations, *k_c _*is the single-site volume-dependent rate constant associated with the mass-action rate law of *R_c_*, *v_c_*(*l_c_, s*) is a function that gives the number of distinct reaction centers in species instance *s *matched by *l_c_*, and  is a function that gives the number of distinct reaction centers in species instance *s *matched by both *l_c _*and . In the case where no species instance is matched by both *l_c _*and , the positive, first term in Eq. 9 accounts for all possible combinations of reactants and the second term reduces to zero. In other cases, the first term over counts the number of combinations, and the second term corrects for the over counting.

On-the-fly updates of rates of the form of *r_c _*are fairly complicated but follow from Eq. 9. Basically, when the system state changes, *r_c _*is incremented or decremented by the terms in Eq. 9 that are added or removed as a result of the system state change.

#### Unimolecular monogamous ring-closure reactions

We will use *R_d _*to denote a rule that defines unimolecular monogamous ring-closure reactions, such as that illustrated in Fig. 1(d). We assume that *R_d _*has the following form: **, where *l_d _*and  are LHS pattern graphs and *ρ *_*d *_is a RHS pattern graph. Because *l_d _*and  are not separated by a plus sign, these pattern graphs can be understood to identify two reactive components within the same chemical species, consistent with the conventions of BNGL [21]. Here, we further assume that these reactive components lie within the same molecule. The pair of components identified by *l_d _*and  forms the reaction center identified by *R_d_*, which defines a graph-rewriting operation that connects the two vertices in the reaction center.

Given a set of species instances  at time *t*, the instantaneous rate *r_d_*(*t*) associated with *R_d _*is given by(12)

where(13)

and(14)

In the above equations, *k_d _*is the single-site rate constant associated with the mass-action rate law of *R_d_*, *v_d_*(*l*_*d*_, *m*) is a function that gives the number of distinct reaction centers within a common molecule *m *in a species instance *s *matched by *l_d_*, and  is a function that gives the number of distinct reaction centers within a common molecule *m *in a species instance *s *matched by both *l_d _*and . In BNGL, the graphs that represent chemical species are composed of graphs that represent types of molecules [21,27]. Thus, *m *in Eq. 12 references a particular subgraph of a species instance graph *s *that corresponds to a type of molecule. Updates of *r_d _*necessitated by system state changes follow from Eq. 12.

#### Unimolecular non-monogamous ring-closure reactions

We will use *R_e _*to denote a rule that defines unimolecular non-monogamous ring-closure reactions, such as that illustrated in Fig. 1(e). We assume *R_e _*has the following form:  where *l_e _*and  are LHS pattern graphs and *ρ *_*e *_is a RHS pattern graph. According to the conventions of BNGL [21], the LHS pattern graphs *le *and  identify two reactive components within the same chemical species. Here, we further assume that these reactive components lie in different molecules within the same chemical species. The pair of components identified by *l_e _*and  forms the reaction center identified by *R_e_*, which defines a graph-rewriting operation that connects the two vertices in the reaction center.

Given a set of species instances  at time *t*, the instantaneous rate *r_e_*(*t*) associated with *R_e _*is given by(15)

where(16)

and(17)

In the above equations, *k_e _*is the single-site rate constant associated with the mass-action rate law of *Re*, *v_e_*(*l*_*e*_, *m*) is a function that gives the number of distinct reaction centers within a common molecule *m *in a species instance *s *matched by *l_e_*, and  is a function that gives the number of distinct reaction centers within a common molecule *m *in a species instance *s *matched by both *l_e _*and . Updates of *r_e _*necessitated by system state changes follow from Eq. 15.

### Usage considerations

In the Actions block of a BioNetGen input file [21], a RuleMonkey-specific command, simulate_-_rm, is used to initiate a simulation. This command takes the same arguments as analogous commands that invoke other simulation engines, such as DYNSTOC [14]. The output of a RuleMonkey simulation is a .info file and a .gdat file. The .info file reports metadata about a simulation run, such as the execution time. The .gdat file reports a time course for each observable quantity defined in an input file (a file with a .bngl extension). Observables are sums or weighted sums of population levels of chemical species (Eq. 3), and they are defined according to the conventions of BNGL [21]. The time points at which results are reported in the .gdat file are specified by a user using the simulate_-_rm command. These results are generated through evaluation of the system state at each of the time points of interest.

## Results

### Validation

**Figure 2:**
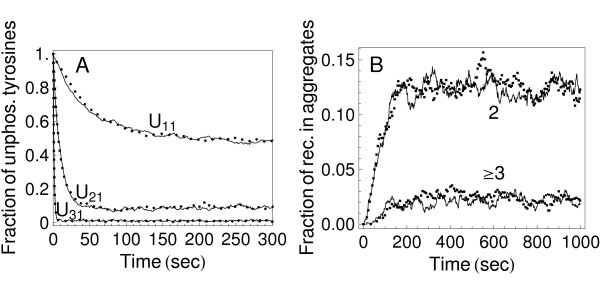
**Example validation results**. a) Simulation of the model of Test Case I of Colvin et al. [14] using DYNSTOC (dotted line) and RuleMonkey (solid line). Parameters used are the same as those reported for Fig. 2A of Colvin et al. [14]. The BioNetGen input file processed by DYNSTOC and RuleMonkey is called testcase1.bngl (Additional file 1). b) Simulation of the TLBR model, the model of Test Case II of Colvin et al. [14], using DYNSTOC (dotted line) and RuleMonkey (solid line). Parameters used are the same as those reported for Fig. 2B of Colvin et al. [14]. The BioNetGen input file processed by DYNSTOC and RuleMonkey is called testcase2b.bngl (Additional file 3).

**Figure 3:**
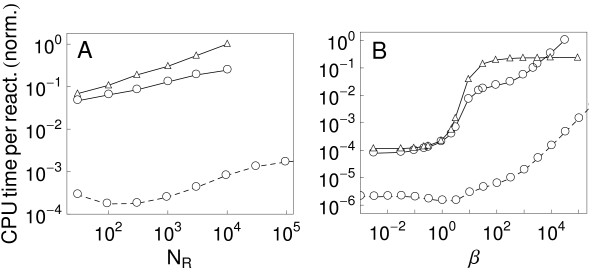
**RuleMonkey is efficient for simulation of rule-based models characterized by large-scale networks**. We compare RuleMonkey (solid lines marked by triangles), DYNSTOC (solid lines marked by open dots) and a problem-specific implementation of the method of Yang et al. [19] (dotted lines); these methods are used to simulate the TLBR model. a) Scaling of computational cost with system size, where size is measured by *N_R_*, the number of cell-surface receptors. b) Scaling of computational cost with dimensionless parameter *β *= *N*_*R*_*k*+2/*k*_off_, which controls the (equilibrium) extent of ligand-induced receptor crosslinking. The rate constant *k*_+2 _characterizes receptor crosslinking, and the rate constant *k*_off _characterizes dissociation of ligand-receptor bonds. The value of *β *was adjusted by varying *k*_+2 _while holding *N*_*R *_= 300 and *k*_off _= 0.01 s^-1 ^fixed. In each panel, the y-axis indicates the normalized total CPU time per reaction event required to simulate the kinetics of the TLBR model from time *t *= 0 to 1000 s with all ligand initially free. Parameters used are the same as those reported for Fig. 3 of Colvin et al. [14]. See the BioNetGen input file testcase2a.bngl (Additional file 2).

**Figure 4:**
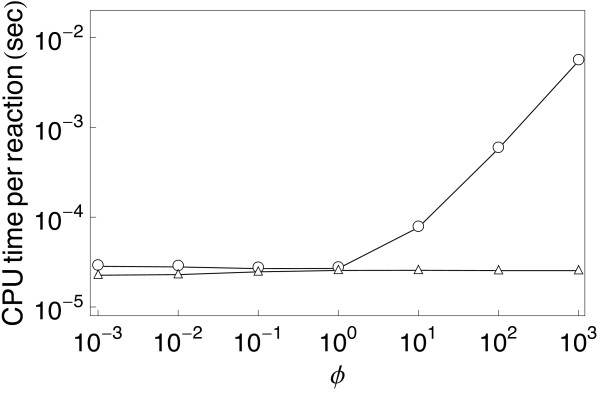
**RuleMonkey is efficient for simulation of networks with fast reactions**. The model considered here is specified in Additional file 4 (stiff.bngl). RuleMonkey (triangles) is compared with DYNSTOC (open dots). The y-axis indicates the total CPU time per reaction event required to simulate the kinetics of two first-order reactions. The x-axis indicates the value of *ϕ*, the ratio between the rate constants that characterize the two reactions. In RuleMonkey, the time step is sampled from an exponential distribution scaled by the total reaction rate (Eq. 5). In contrast, in DYNSTOC, the time step is fixed and limited by the rate of the fastest reaction [14]. This difference in how the time step is selected accounts for the performance differences seen for cases where *ϕ *≫ 1.

We validated RuleMonkey by comparing simulation results against those obtained using BioNetGen [25,29], DYNSTOC [14], and problem-specific codes [19,28,44]. The following models were considered (Table 1; Figs. 2, 3, 4): 1) the multisite phosphorylation model introduced by Colvin et al. [14], testcase1.bngl (see Additional file 1); 2) the TLBR (trivalent ligand-bivalent receptor) model introduced by Yang et al. [19] and considered by Colvin et al. [14] and here in Fig. 2B, testcase2a.bngl (see Additional file 2) and testcase2b.bngl (see Additional file 3); 3) a model introduced here with reaction events occurring on disparate time scales, stiff.bngl (see Additional file 4); 4) a model introduced here for interaction of a pentavalent ligand with a trivalent cell-surface receptor, pltr.bngl (see Additional file 5); 5) the model of Blinov et al. [45] for early events in epidermal growth factor receptor (EGFR) signaling, egfr net.bngl (see Additional file 6); 6) the model of Goldstein et al. [46] and Faeder et al. [47] for early events in IgE receptor (FcϵRI) signaling, fceri.bngl (see Additional file 7); and 7) the model of Nag et al. [44] for crosslinking of phosphorylated LAT molecules by intracellular Grb2-Sos1 complexes, lat.bngl (see Additional file 8). The BioNetGen input files that specify these models and RuleMonkey simulations of them are available as Additional files 1,2,3,4,5,6,7,8. They are also available at the RuleMonkey web site [40].

**Table 1:** Relative efficiency of RuleMonkey for benchmark problems

Bench- mark	Input file (.bngl)	Reference(s)	RuleMonkey	DYNSTOC	Problem specific code	BioNetGen
1	testcase1	[14]	1.3 × 10^-5^	1.6 × 10^-2^	N/A	3.4 × 10^-5^
2	testcase2b	[14,19]	2.4 × 10^-5^	1.2 × 10^-4^	2.2 × 10^-5^	--
3	stiff	This study	4.6 × 10^-6^	2.6 × 10^-4^	N/A	5.0 × 10^-7^
4	pltr	[20]	1.1	1.1	4.1 × 10^-5^	--
5	egfr net	[45]	1.1 × 10^-5^	1.8 × 10^-4^	N/A	3.7 × 10^-6^
6	fceri	[46,47]	1.9 × 10^-5^	1.9 × 10^-3^	N/A	6.7 × 10^-6^
7	lat	[44]	9.9 × 10^-3^	1.3 × 10^-2^	1.8 × 10^-5^	--

The performance of RuleMonkey for seven benchmark problems is compared against that of DYNSTOC [14], problem-specific codes implementing network-free procedures [20,44], and the simulate_-_ssa procedure of BioNetGen [21,25,29]. The problem-specific codes for benchmark problems 2, 4 and 7 were provided by M. I. Monine; these codes have been described by Yang et al. [19], Monine et al. [28], and Nag et al. [44]. Each table entry indicates seconds of CPU time per reaction event. For benchmark problems 2, 4, and 7, BioNetGen is unable to exhaustively generate the reaction network needed for generate-first simulation (without network truncation), as these problems involve simulating polymerization-like reactions. For BioNetGen simulations, the cost of network generation is not included in the table entries. All simulations were performed on a Macintosh desktop computer, equipped with a single G5 processor. Simulations were performed as specified in the indicated BioNetGen input files, which are available as Additional files 1 and 3-8 and at the RuleMonkey web site [40]. The benchmark problems are described in more detail in the text and in the references cited.

Each model is specified as a BioNetGen input file, which can be processed by BioNetGen, DYNSTOC, and RuleMonkey. The input files for these different software tools are the same, except for the simulation commands in the Actions blocks [21]. As noted above, the simulation command for RuleMonkey is simulate_-_rm. BioNetGen accepts two simulation commands, simulate_-_ode and simulate_-_ssa. In calculations with BioNetGen, we used simulate_-_ssa, which invokes an efficient extension of Gillespie's method, preceded by a generate_-_network command, which prompts BioNetGen to implement the procedures needed to explicitly derive the reaction network implied by a set of rules. In other words, we only used BioNetGen to perform simulations via the so-called generate-first approach [9,21,48].

The reason we only consider generate-first simulations using BioNetGen is that simulation cost and network-generation cost can be easily separated when this simulation approach is taken, as it consists of two steps. The first step is network generation. The second step is simulation. In the on-the-fly approach, execution switches back and forth between network generation and simulation. It has been observed that stochastic simulation via the on-the-fly approach tends to be faster than stochastic simulation via the generate-first approach [35]. Thus, if we find that generate-first simulation is faster than network-free simulation, we can assume with some confidence that on-the-fly simulation is also faster than network-free simulation.

Typical validation results are shown in Fig. 2. In all cases, RuleMonkey was found to produce results consistent with those obtained using the other methods/codes considered.

### Performance

As can be seen from the results of Table 1, RuleMonkey is more efficient than DYNSTOC, but less efficient than BioNetGen and problem-specific codes (for cases where BioNetGen and problem-specific codes can be applied). BioNetGen is unable to simulate three of the test models via the generate-first approach because the reaction networks implied by the rule sets of these models cannot be generated exhaustively. Thus, of the available general-purpose simulation codes compliant with the conventions of BNGL, RuleMonkey and DYNSTOC are the most widely applicable (because these tools implement general-purpose network-free simulation methods), and RuleMonkey is more efficient than DYNSTOC (Table 1). It should be noted that we would expect the generate-first approach to simulation [9,21,48], which may involve either stochastic simulation or numerical integration of ODEs (although we consider only stochastic simulation here), to be more efficient than network-free simulation for cases where the generate-first approach is feasible because of the bookkeeping costs of network-free simulation, which requires data structures and update schemes to track the component state of a system. Unfortunately, simulation methods that rely on derivation of the reaction network implied by a rule set, which is computationally expensive, are often not feasible for rule sets that imply large-scale chemical reaction networks. Feasibility depends on effective network size, which is a function of model parameter values. For some parameter values, network generation can be halted arbitrarily or limited as in the on-the-fly approach to simulation [9,21,48]. For other parameter values, as demonstrated by Yang et al. [19], for example, network generation is a limiting factor, and network-free simulation is essential.

The performance of RuleMonkey scales with problem size in a way that is similar to that of DYNSTOC [14] and the problem-specific codes of Yang et al. [19] and Yang and Hlavacek [20]. Results obtained from simulation of the TLBR model of Yang et al. [19] are shown in Fig. 3. The TLBR model, a kinetic version of the model of Goldstein and Perelson [49], characterizes the interactions of trivalent ligands with bivalent cell-surface receptors. In Fig. 3(a), the cost of simulation is shown as a function of the number of receptors *N_R_*. As *N_R _*increases, the frequency of reactions increases. In Fig. 3(b), the cost of simulation is shown as a function of the dimensionless parameter *β*, which characterizes ligand-induced receptor crosslinking at equilibrium. As *β *increases, the equilibrium size of ligand-induced receptor aggregates increases. Thus, this parameter effectively controls the number of populated species and the number of reactions with non-zero flux. As can be seen, the cost of simulation is constant up to a critical value of *β*, where a percolation transition occurs [19,49]. Above this threshold, simulation cost increases linearly. Yang et al. [19] have shown that this scaling arises because of the expense of enforcing an application condition of a rule of the TLBR model that prohibits the formation of cyclic receptor aggregates. Thus, in the case of the TLBR model, simulation efficiency is affected by model parameter values. We expect that simulation of the model of Benchmark Problem 4 in Table 1, which is closely related to the TLBR model, is affected by model parameter values in a similar way.

Finally, we compared the performances of RuleMonkey and DYNSTOC for problems with reactions occurring on different time scales (Fig. 4). As can be seen, as the rate of the fastest reaction in a system increases, the efficiency of DYNSTOC degrades relative to that of RuleMonkey.

## Conclusions

RuleMonkey is a general-purpose simulator for rule-based models encoded in BNGL. RuleMonkey complements BioNetGen [21,25,29], the first software tool to have the capability to simulate rule-based models encoded in BNGL. The rules that comprise BNGL-encoded models can be expanded by BioNetGen into the set of reactions implied by the rules, and BioNetGen can simulate the kinetics of these reactions using conventional methods, such as numerical integration of the corresponding system of ordinary differential equations (ODEs) for mass-action kinetics. Although BioNetGen has been used to study a number of systems [22-24,45,50], the reaction network implied by a set of rules is often so large as to make the simulation approaches implemented in BioNetGen impractical. Network generation is expensive, as is simulation of a large-scale reaction network using a conventional method. Such methods have a computational cost that depends on the size of the network being simulated. In the generate-first and on-the-fly approaches to simulating rule-based models [9,21,25,35,48], which are implemented in BioNetGen, there is both a network generation cost and a network simulation cost, because network generation is a prerequisite for network simulation via these approaches. In contrast, in network-free simulation approaches [14,15,18-20], such as the method presented here and implemented in RuleMonkey, there is only a network simulation cost. However, it should be noted that network-free simulation procedures have a relatively high cost per reaction event, as is evident in Table 1. Thus, one should choose such a simulation procedure only when the cost of an alternative procedure that relies on network-generation is prohibitive.

The cost of network generation is often prohibitive, which has motivated the development of several network-free simulation methods [14,15,18-20]. Like these methods, the method presented here and implemented in RuleMonkey avoids network generation and, consequently, has a computational cost independent of the number of reactions implied by a set of rules (Figs. 2 and 3). We note that Yang and Hlavacek [20] have described a method that is essentially the same as that implemented in RuleMonkey but without providing details about how the method could be implemented for general use (i.e., beyond problem-specific demonstrations of the method) and without providing general-purpose software.

RuleMonkey and DYNSTOC [14] are similar in that they can both simulate BNGL-encoded models while avoiding network generation. However the method implemented in RuleMonkey is a rejection-free method, whereas the method implemented in DYNSTOC is a null-event method. Both types of methods are capable of producing correct simulation results but null-event methods tend to have a higher cost for simulation of systems with fast reactions [51]. For cases that we have examined, RuleMonkey typically performs better than DYNSTOC (Table 1) and has a decided advantage for systems with fast reactions (Fig. 4). This advantage is perhaps significant because practical problems tend to involve reactions occurring on disparate time scales.

The capability of RuleMonkey to correctly process BNGL-encoded model and simulation specifications (as illustrated in Fig. 2) is significant in that this capability allows RuleMonkey to be used to simulate a wide array of models that account for the site-specific details of protein-protein interactions (e.g., multisite phosphorylation) as well as the connectivity of proteins in protein complexes (e.g., cyclic aggregates of proteins can be distinguished from acyclic aggregates). RuleMonkey also distinguishes between intra- and intermolecular reactions, which is important for correct simulation results in many cases [19,28]. We have demonstrated that RuleMonkey can be used to efficiently simulate rule sets that imply large-scale chemical reaction networks (Table 1; Figs. 2 and 3). The capabilities of RuleMonkey complement those of BioNetGen and other available software tools for simulation of rule-based modeling [14,35,52-56]. In the future, we expect RuleMonkey will prove useful for simulating models of signal-transduction systems that are significantly larger and more comprehensive than the rule-based models of such systems that have so far been considered [22-24,45-47].

## Availability and requirements

• **Project name**: RuleMonkey

• **Project home page**: http://public.tgen.org/rulemonkey

• **Operating system(s)**: Platform independent

• **Programming language**: C

• **Other requirements**: None

• **License**: GNU General Public License (GPL), version 3

• **Any restrictions to use by non-academics**: If the terms of the GPL are unacceptable, it may be possible to provide the software under a different license.

## Authors' contributions

JC implemented the algorithm and designed the tool. MIM and RNG tested and validated the tool and evaluated its performance. DVH, RGP and WSH planned the project. RGP and WSH conceived the tool and wrote the manuscript. All authors have read and approved the final version of the manuscript.

## Supplementary Material

Additional file 1 **This plain-text file specifies a simulation of the multisite phosphorylation model introduced by Colvin et al**. [14]. This simulation is considered in Fig. 2A and Table 1. The file can be processed by RuleMonkey and other BNGL-compatible tools for simulating rule-based models. Click here for file

Additional file 2 **This plain-text file specifies a simulation of a model for trivalent ligand-bivalent cell-surface receptor interaction (the so-called TLBR model) introduced by Yang et al**. [19]. This simulation is considered in Fig. 3. The file can be processed by RuleMonkey and other BNGL-compatible tools for simulating rule-based models. Click here for file

Additional file 3 **This plain-text file specifies a simulation of a model for trivalent ligand-bivalent cell-surface receptor interaction (the so-called TLBR model) introduced by Yang et al**. [19]. This simulation is considered in Fig. 2B and Table 1. The difference between the files testcase2a.bngl and testcase2b.bngl is in parameter values. The file can be processed by RuleMonkey and other BNGL-compatible tools for simulating rule-based models. Click here for file

Additional file 4 **This plain-text file specifies a simulation of reaction events occurring on disparate time scales**. This simulation is considered in Fig. 4 and Table 1. The file can be processed by RuleMonkey and other BNGL-compatible tools for simulating rule-based models. Click here for file

Additional file 5 **This plain-text file specifies a simulation of a model for pentavalent ligand-trivalent cell-surface receptor interactions**. The model is based on assumptions similar to those upon which the TLBR model [19] and the model of Goldstein and Perelson [49] are based. This simulation is considered in Table 1. The file can be processed by RuleMonkey and other BNGL-compatible tools for simulating rule-based models. Click here for file

Additional file 6 **This plain-text file specifies a simulation of the model of Blinov et al**. [45] for early events in epidermal growth factor receptor (EGFR) signaling. This simulation is considered in Table 1. The file can be processed by RuleMonkey and other BNGL-compatible tools for simulating rule-based models. Click here for file

Additional file 7 **This plain-text file specifies a simulation of the model of Goldstein et al**. [46] and Faeder et al. [47] for early events in IgE receptor (FcϵRI) signaling. This simulation is considered in Table 1. The file can be processed by RuleMonkey and other BNGL-compatible tools for simulating rule-based models. Click here for file

Additional file 8 **This plain-text file specifies a simulation of the model of Nag et al**. [44] for crosslinking of phosphorylated LAT molecules by intracellular Grb2-Sos1 complexes. This simulation is considered in Table 1. The file can be processed by RuleMonkey and other BNGL-compatible tools for simulating rule-based models. Click here for file
